# Diagnostic and Prognostic Values of Serum EpCAM, TGM2, and HE4 Levels in Endometrial Cancer

**DOI:** 10.3389/fonc.2020.01697

**Published:** 2020-09-04

**Authors:** Ting Lan, Chunyan Mu, Zhongcheng Wang, Yue Wang, Ying Li, Yueqin Mai, Shibao Li, Hao Xu, Bing Gu, Lan Luo, Ping Ma

**Affiliations:** ^1^Xuzhou Key Laboratory of Laboratory Diagnostics, Xuzhou Medical University, Xuzhou, China; ^2^School of Medical Technology, Xuzhou Medical University, Xuzhou, China; ^3^Department of Laboratory Medicine, Affiliated Hospital of Xuzhou Medical University, Xuzhou, China; ^4^Air Force Jinan Base Security Department Outpatient Department, Jinan, China; ^5^Department of Obstetrics and Gynecology, Affiliated Hospital of Xuzhou Medical University, Xuzhou, China

**Keywords:** endometrial cancer, EpCAM, TGM2, HE4, diagnosis, prognosis

## Abstract

**Objectives:** This study aims to investigate the diagnostic and prognostic values of EpCAM, TGM2, and HE4 in endometrial cancer (EC).

**Methods:** In this study, 42 patients diagnosed with EC (EC group), 41 patients diagnosed with myoma (benign group), and 43 healthy women (healthy group), who applied to Affiliated Hospital of Xuzhou Medical University between March 2018 - September 2019 were recruited. Serum EpCAM, TGM2, and IL-33 levels were measured by ELISA, while serum HE4 and CA-125 levels were measured by ECLIA. The serum markers listed above were also measured in 12 paired pre- and post-operative EC patients. The diagnostic and prognostic values of serum markers were analyzed.

**Results:** The serum EpCAM, TGM2, HE4, CA-125, and IL-33 levels were significantly higher in the EC group. The sensitivity and specificity of combined detection of EpCAM and HE4 was 92.86 and 69.05%, which were significantly higher than using a single marker or other combinations. Among these markers, serum HE4 levels were significantly higher in patients with myometrial invasion, metastasis, and lymphovascular invasion (*p* = 0.006, *p* = 0.0004, *p* = 0.0004, respectively). And serum TGM2 levels were significantly decreased in post-operative than that of pre-operative EC patients (*p* < 0.001).

**Conclusions:** The combination of EpCAM and HE4 showed the highest specificity and sensitivity in the diagnosis of EC. HE4 was successful in the detection of high-risk individuals preoperatively. Additionally, TGM2 might be a prognostic factor for EC.

## Introduction

Endometrial cancer (EC) is the most common gynecologic malignancy worldwide. In 2018, the incidence and mortality of EC have been remarkably rising with an estimated 382, 069 new cases and 89, 929 deaths globally (1). Approximately 90% of EC patients presents abnormal vaginal bleeding, allowing early detection and prevention (2). However, other EC patients with atypical symptoms usually result in delayed diagnosis and treatment, and ultimately poor prognosis. Notably, EC patients diagnosed at stage I indicate a high 5-year survival rate of 95%, while it declines to 69%, and 17% for stage III and IV, respectively (3). Moreover, 10–15% of EC patients with stage I will undergo a recurrence within 3 years after therapy (4). Therefore, early detection and follow-up are pivotal to heighten the survival rate of EC patients.

Current common tests for EC diagnosis include ultrasound, hysteroscopy, pelvic examination, endometrial biopsy, dilation, and curettage. Among which, endometrial biopsy or dilation and curettage helps to classify EC stages but is an invasive procedure accompanied by pain or cramp, hemorrhage, infection, and rarely uterine perforation (5). Patients with abnormal vaginal bleeding will have to receive endometrial biopsy to exclude cancer, though metrorrhagia also happens in many other benign diseases (6, 7). Thus, non-invasive diagnostic methods such as blood biomarkers would be preferable for clinical practice. Indeed, recent emerging work has identified numerous potential biomarkers for early diagnosis and prognosis of EC (8). Unfortunately, none of these suggested biomarkers have been recommended for routine EC evaluation, screening, and monitoring.

To date, the well-investigated biomarkers for EC evaluation are cancer antigen 125 (CA-125) and human epididymis protein 4 (HE4). Multiple studies have demonstrated that both CA-125 and HE4 have potential prognostic properties (9–13). While, in comparison to CA-125, HE4 reveals a superior diagnostic accuracy to distinguish physiological, benign, or malignant gynecological diseases (9, 14). It is worth noting that elevated CA-125 and HE4 levels are also detected in other non-gynecological diseases (15, 16). Nevertheless, CA-125 and HE4 are frequently employed as reference standards for novel biomarkers discovery.

Recently, many new emerging biomarkers such as epithelial cell adhesion molecule (EpCAM), transglutaminase 2 (TGM2), and interleukin-33 (IL-33) have also shown their potentials for EC evaluation. EpCAM is highly expressed in endometrial tissues as an epithelial marker and is frequently overexpressed in EC (17, 18). TGM2, a cancer cell survival factor in multiple tumor types, presents a good EC detection property (19). IL-33, a cytokine involved in the regulation of anti-tumor immunity and tumor growth, its levels are significantly higher in EC patients than that of healthy volunteers (20, 21). However, due to the small number of endometrial biopsies, routine usage of EpCAM, TGM2, and IL-33 could not be established for EC diagnosis.

In this study, we aimed to determine the efficacy of EpCAM, TGM2, HE4, CA-125, and IL-33 in the diagnosis and prognosis of EC.

## Materials and Methods

### Eligibility Criteria

Participants were consecutively enrolled in this prospective observational study conducted in the Affiliated Hospital of Xuzhou Medical University in March 2018 - September 2019. This study was approved by the Ethics Committee of the Affiliated Hospital of Xuzhou Medical University (XYFY2019-KL113-01). All participants provided written informed consent.

Inclusion criteria were as follows: (1) women that were pathologically diagnosed with EC were included in the EC group; (2) women that were pathologically diagnosed with uterine myoma were included in the benign group; (3) women with normal findings both at physical and color doppler ultrasound examinations were included in the healthy group.

Exclusion criteria were as follows: (1) history of other primary or secondary tumors; (2) history of other major comorbidities (e.g., hypertension, diabetes, heart disease, renal disease, etc.); (3) pre-operative chemotherapy or radiotherapy; (4) hormonal treatment before surgery.

**Figure 1:**
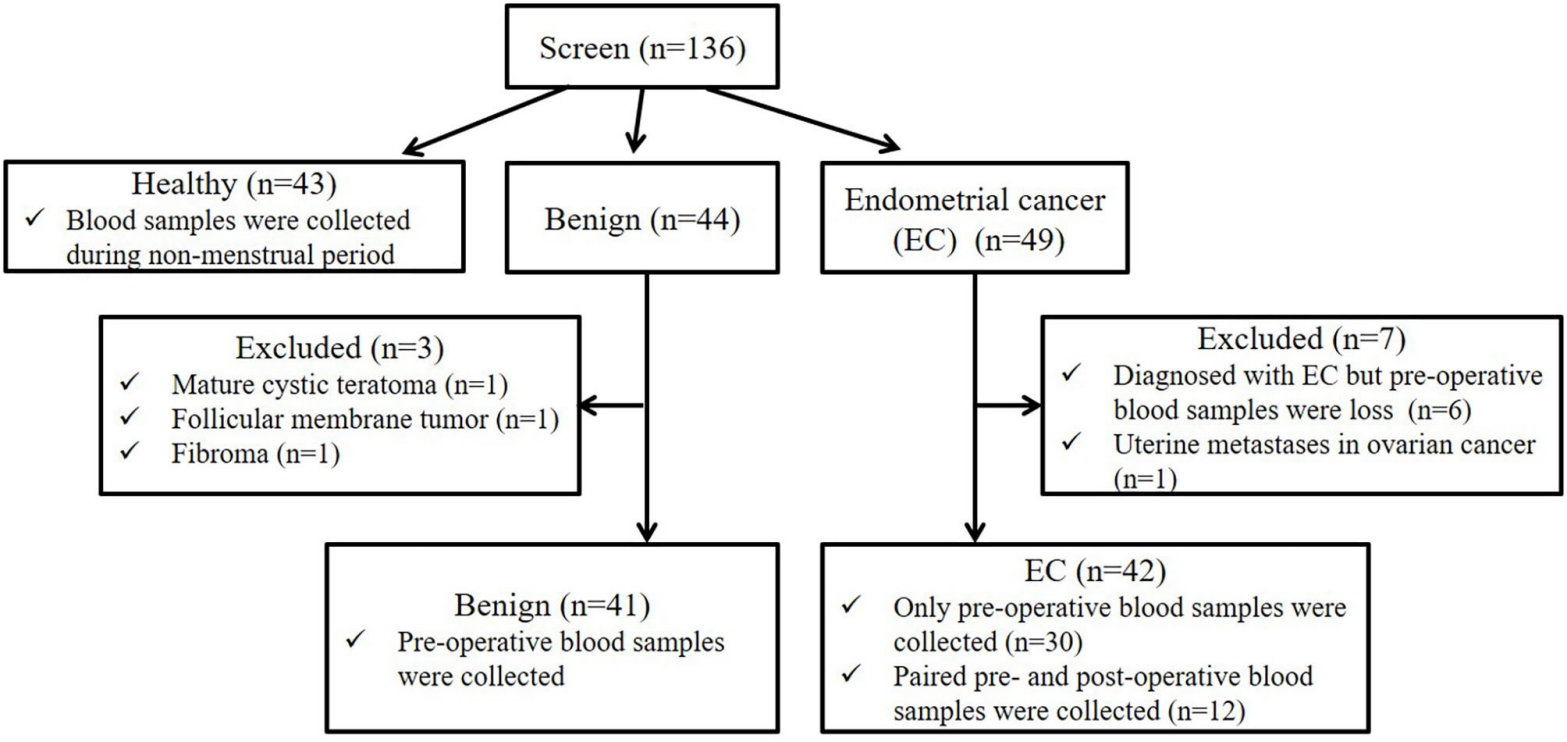
Participant screening flow chart. We screened 136 participants, including 43 healthy women, 44 benign and 49 EC patients. Among 44 benign patients, 3 patients with other tumors were excluded. Among 49 EC patients, 6 EC patients lacked pre-operative blood samples, and 1 patients with metastatic EC were excluded. Among 42 patients included in the EC group, paired pre- and post-operative blood samples were obtained from 12 patients.

In this study, a total of 136 participants were screened (the flow chart for enrollment was shown in Figure 1). Among them, 43, 44, and 49 participants were included in the healthy, benign, and EC group, respectively. All patients in the benign and EC group underwent surgery and were diagnosed based on post-operative histology. For the healthy group, blood samples were obtained during the non-menstrual period. For the benign and EC group, blood samples were obtained on the day before surgery. In the benign group, 41 patients that were pathologically diagnosed with uterine myoma were included, 1 patient with mature cystic teratoma, 1 patient with follicular membrane tumor, and 1 patient with fibroma were excluded. In the EC group, 42 patients that were pathologically diagnosed with EC were included, 6 patients diagnosed with EC but lacked pre-operative blood samples and 1 patient with metastatic EC were excluded. Among patients included in the EC group, paired pre- and post-operative blood samples were obtained from 12 patients. EC patients were staged according to the International Federation of Gynecology and Obstetrics (FIGO) surgical/pathological staging guidelines (22).

### Detection Methods

Five milliliter blood samples from elbow vein were collected and placed in a yellow test tube (Guangzhou Yangpu Biotechnology Co., Ltd., Guangzhou, China). After standing for 10 min, the samples were centrifuged at 3,500 rpm/min for 10 min at 4°C. Then the supernatant was added to the sterile EP tube (Jiangsu Kangjian Biotechnology Co., Ltd., Jiangsu, China) and stored in the refrigerator at −80°C. Serum TGM2 and IL-33 levels were detected by ELISA Kit (Shanghai Tongwei Industrial Co., Ltd, Shanghai, China). Serum EpCAM level was detected by ELISA Kit (Beijing Biotyscience Biology Technology Co., Ltd, Beijing, China). Serum CA-125, HE4, triglyceride (TG), total cholesterol (TC) glucose (GLU), low-density lipoprotein (LDL), high-density lipoprotein (HDL) were detected by ECLIA from Roche COBASE 601 automatic chemiluminescence instrument (Roche Pharmaceutical Ltd, Shanghai, China) and original matching reagent. Moreover, all procedures were performed according to the kit instructions, and all controls were within the scope stated in the instructions.

### Positive Criteria

In this study, the levels of TGM2>359.71 pg/ml, IL33>1.29 pg/ml, and EpCAM>204.90 pg/ml were defined as positive according to the ROC (receiver operating characteristic) curves, respectively. The levels of CA-125>35.00 U/mL, and HE4>92.10 pmol/l (premenopausal) or HE4>121.00 pmol/l (postmenopausal) were defined as positive based on the reference range provided by the kit.

### Statistical Analysis

All the data were analyzed using IBM SPSS Statistics (Version 24) and GraphPad Prism software (Version 6.01). Categorical data were evaluated using the Normality test. If *p* ≥ 0.1, LSD was carried out. If the data is not normally distributed, the Mann-Whitney *U*-test was chosen while *p* < 0.05 in the Kruskal-Wallis test. Count data were evaluated between groups using the *x*^2^-test. Paired-samples *T*-test was executed between paired samples. The measurement data were expressed as the means of the data ± standard deviation (SD) if the data is normally distributed. Otherwise, median and minimum to maximum were used to describe the date. The ROC curves and AUC (area under the curve) were used to assess the diagnostic value of EpCAM, TGM2, HE4, CA-125, and IL-33. When the Youden index is maximum, the cutoff values of EpCAM, TGM2, and IL-33 are determined. *p* < 0.05 was accepted as statistically significant.

## Results

### Demographic Parameters and Clinical Features in Each Group

The median age in the healthy, benign, and EC group was 47 years (range 40–59), 46 years (range 22–56), and 52.5 years (range 31–77), respectively. Overweight and obesity are demonstrated to be linked to approximately one-third of EC cases (23). Herein, we analyzed the serum TG, TC, GLU, LDL, HDL levels, and diabetes in each group. The serum TG levels were significantly increased in the EC group as compared to the healthy (*p* = 0.001, Table 1) and benign group (*p* = 0.007, Table 1). However, the significant declined serum HDL levels were observed in the EC group when compared with the healthy group (*p* = 0.032, Table 1) but not the benign group (*p* = 0.425, Table 1). For other markers, there were no substantial differences between each group (Table 1). And the percentage of patients diagnosed with diabetes was not significantly different among the benign and EC group (Table S1). These results demonstrated that obesity was correlated with the occurrence of EC, which coincided with previous reports (24, 25). And diabetes was not a risk factor for EC.

**Table 1:** Comparison of demographic parameters, medical history, and clinical features in endometrial cancer group, benign group, and healthy group.

	**Healthy (*n* = 43)**	***p^***a***^***	**Benign (*n* = 41)**	***p^***b***^***	**EC (*n* = 42)**
	**Median (min-max)/Mean ± SD**		**Median (min-max)/Mean ± SD**		**Median (min-max)/Mean ± SD**
Age (years old)	47.00 (40.00–59.00)	**0.002**	46.00 (22.00–56.00)	**<0.001**	52.50 (31.00–70.00)
EpCAM (pg/ml)	187.66 (111.68–280.47)	**<0.001**	199.16 (69.38–381.70)	**0.004**	241.22 (133.39–380.97)
TGM2 (pg/ml)	473.64 (0–6,300.00)	0.429	369.80 (0–5,010.23)	**<0.001**	500.72 (213.72–6,040.93)
IL-33 (pg/ml)	2.22 (0–68.80)	**0.004**	7.02 (83–84.00)	0.466	4.65 (0.30–69.91)
CA125 (ng/ml)	12.08 (5.36–50.96)	**<0.001**	15.06(6.37-81.94)	**0.004**	20.73 (6.84–147.70)
HE4 (pmol/ml)	32.51 (21.21–48.88)	**<0.001**	46.26 (32.09–75.54)	**0.001**	58.54 (30.98–455.40)
TG (mmol/L)	1.16 (0.60–1.92)	**0.001**	1.12 (0.64–16.00)	**0.007**	1.51 (0.55–4.11)
TC (mmol/L)	5.02 (3.41–6.03)	0.961	4.81 (2.89–7.14)	0.125	4.74 (2.49–7.80)
GLU (mmol/L)	5.83 (5.09–6.67)	0.993	5.42 (4.02–10.73)	0.055	5.80 (4.09–14.40)
HDL (mmol/L)	1.43 ± 0.33	**0.032**	1.22 ± 0.36	0.425	1.27 ± 0.30
LDL (mmol/L)	2.87 ± 0.59	0.334	2.47 ± 0.85	0.170	2.70 ± 0.87

*The statistical analysis of EpCAM, TGM2, IL-33, CA125, HE4, TG, TC, and GLU are tested by Kruskal-Wallis. The statistical analysis of HDL and LDL are tested by LSD-t. p < 0.05 is considered as statistically significant. p^a^, Healthy vs. EC; p^b^, Benign vs. EC*.

Furthermore, we compared other demographic features between the benign and EC group that the menopausal status, menarche, term, abortion, and parity were significantly different among the benign and EC group (Table S1).

### Estimation of EpCAM, TGM2, HE4, CA-125, and IL-33 in the Diagnosis of EC

**Figure 2:**
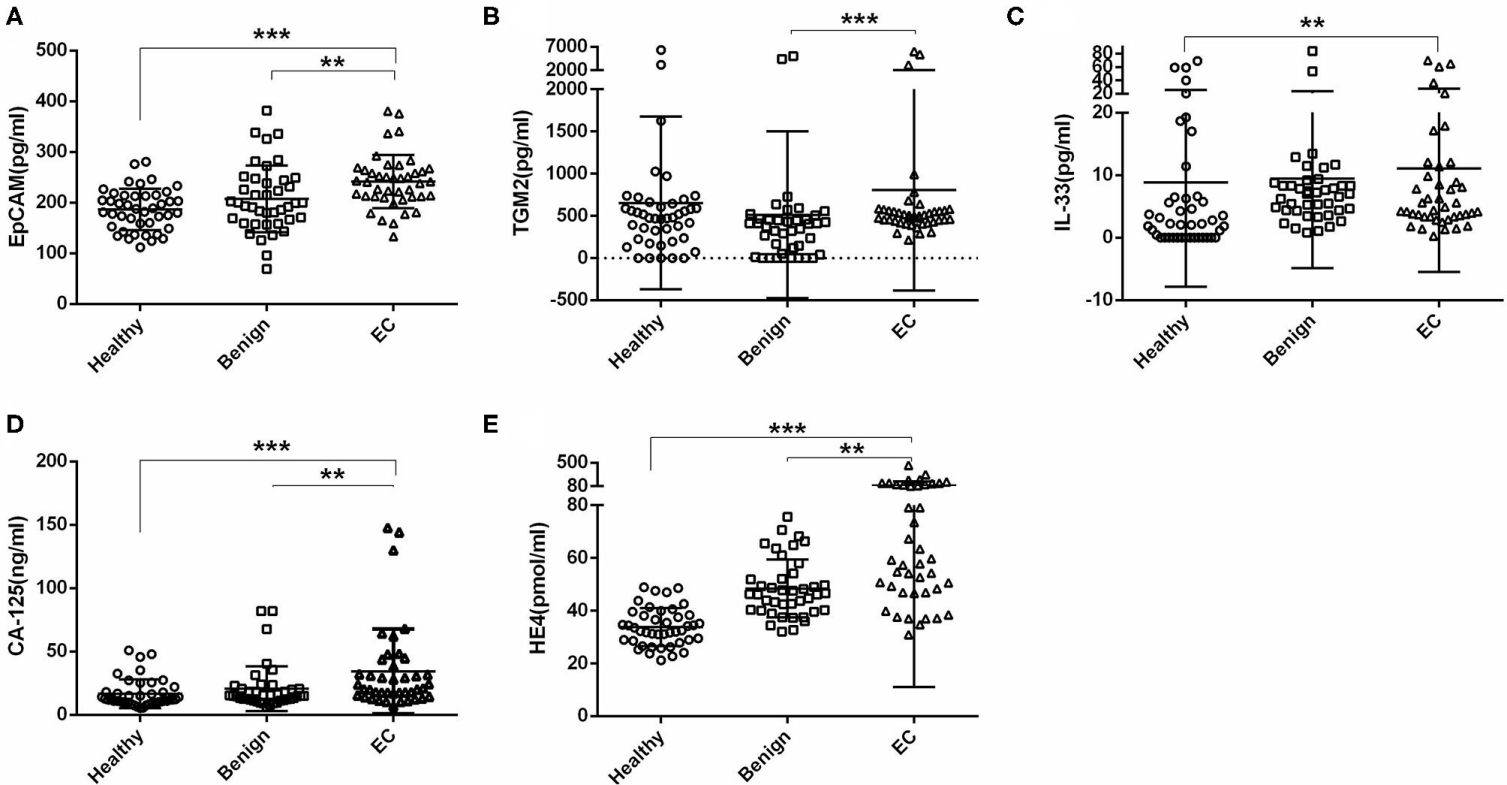
Comparisons of serum EpCAM, TGM2, IL-33, CA-125, and HE4 levels in the healthy, benign, and endometrial cancer (EC) group. **(A)** EpCAM. **(B)** TGM2. **(C)** IL-33. **(D)** CA-125. **(E)** HE4. ***p < 0.001, **p < 0.01.

We first tested the serum EpCAM, TGM2, HE4, CA-125, and IL-33 levels in each group. The median value of EpCAM, CA-125, and HE4 were significantly higher in the EC group as compared to the healthy and benign group (*p* < 0.01, Figures 2A,D,E). The serum TGM2 levels in the EC group were significantly increased as compared to the benign group (*p* < 0.001, Figure 2B, Table 1), but there was no statistical significance when compared to the healthy group (*p* = 0.429, Figure 2B, Table 1). And the serum IL-33 levels were significantly increased in the EC group than that of the healthy group (*p* = 0.004, Figure 2C, Table 1), but it showed no significant difference between the benign and EC group (*p* = 0.466, Figure 2C, Table 1).

**Figure 3:**
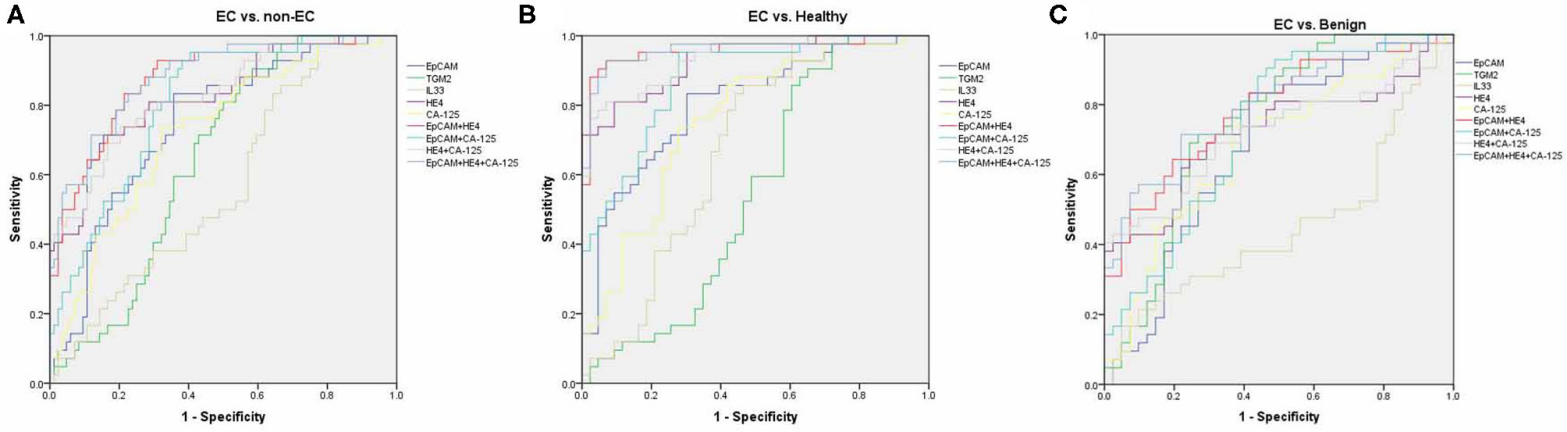
Receiver operating characteristic (ROC) curves. ROC curves for regression models using serum EpCAM, TGM2, IL-33, CA-125, HE4, EpCAM+HE4, CA-125+HE4, EpCAM+CA-125, and EpCAM+CA-125+HE4 to distinguish EC from non-EC **(A)**, healthy **(B)**, and benign group **(C)**, respectively.

By using ROC analysis, we then analyzed the diagnostic values of serum EpCAM, TGM2, HE4, CA-125, and IL-33 in discriminating EC from the healthy and benign group (non-EC group), or individually. To discriminate EC from non-EC (Figure 3A, Table S2), The HE4 exhibited the highest AUC value (0.827) when 49.32 pmol/L was taken for cut-off value (sensitivity of 71%, specificity of 84%). And the EpCAM and CA-125 exhibited relative high AUC values (0.745, 0.722, respectively), but lower than that of HE4. The TGM2 and IL-33 exhibited higher sensitivity over 90% (90.48%, 97.62%, respectively), but lower specificity <50% (41.67%, 22.62%, respectively), making them had low AUC values. Similarly, the HE4 also exhibited the best diagnostic potency in distinguish EC from the healthy group (Figure 3B, Table S3), while the TGM2 was the best biomarker for discriminating EC from the benign group (Figure 3C, Table S4).

We then further tested if the diagnostic accuracy of discriminating EC from non-EC would improve when combined with the two or three biomarkers (EpCAM, CA-125, and HE4, Figure 3A, Table S2). Within the four combinations, the 3-marker model exhibited the highest AUC value of 0.881 (sensitivity of 83.33%, specificity of 77.38%). The EpCAM+HE4 model exhibited a slightly lower AUC value of 0.874 but displayed higher sensitivity (92.86%) than that of the 3-marker model (83.33%), making it the best selection for EC diagnosis. Nevertheless, the EpCAM+HE4 model was also regarded as the best combination for discriminating EC from healthy (Figure 3B, Table S3) or benign patients (Figure 3C, Table S4).

To estimate the potential of serum EpCAM, TGM2, HE4, CA-125, and IL-33 levels in discrimination of different staged EC patients from the non-EC, separate ROC analysis were performed. The models that exhibited the highest AUC value to distinguish stage I, II, or III EC from non-EC were EpCAM+HE4 (0.911, Table S5), EpCAM+CA-125 (0.899, Table S6), EpCAM+CA125+HE4 (0.902, Table S7) model, respectively. Although the EpCAM+CA125+HE4 model displayed the highest AUC value of 0.898 to distinguished stage I and II EC from non-EC, it had lower sensitivity than that of EpCAM +HE4 model (Table S8). Moreover, these models revealed similar diagnostic values when distinguishing EC from healthy or benign patients alone (Tables S9–S16). These data suggested that the EpCAM+HE4 model had the best early diagnostic value for EC patients especially for stage I EC.

### Estimation of EpCAM, TGM2, HE4, CA-125, and IL-33 in the Prognosis of EC

Then we also analyzed the correlation of serum EpCAM, TGM2, HE4, CA-125, and IL-33 levels with clinicopathological factors in the EC group (Table 2). In comparison to young patients (≤50 years old), serum HE4 levels were significantly increased in old patients (>50 years old, *p* = 0.004), suggesting that HE4 might be a critical biomarker for EC diagnosis in old patients. For histological grades, there was no significant difference among any markers we tested. We also classified the stages as early (well or moderately differentiated G1+G2) and late (poorly differentiated G3), and estimated the preoperative serum levels among two groups. Accordingly, there was no significant difference between the two groups for preoperative serum levels of EpCAM, TGM2, HE4, and IL-33 (*p* = 1.000, *p* = 0.408, *p* = 0.857, *p* = 0.432, respectively). Only CA-125 level was found decreased noticeably at a late stage (median of early-stage 24.01 ng/ml; median of late-stage 15.09 ng/ml) and that decreases were statistically significant (*p* = 0.028). We also estimated the connection between markers and prognostic factors including myometrial invasion and metastasis. Among these markers, only serum HE4 levels were found different noticeably in patients with myometrial invasion and metastasis (*p* = 0.006, *p* = 0.004, respectively).

**Table 2:** EpCAM, TGM2, HE4, CA-125, and IL-33 measured in serum samples from 42 patients with endometrial cancer concerning clinicopathological factors.

		**EpCAM (pg/ml)**		**TGM2 (pg/ml)**		**IL-33 (pg/ml)**		**CA-125 (ng/ml)**		**HE4 (pmol/ml)**	
	***N* (%)**	**Median (min-max)/**	***p***	**Median (min-max)/**	***p***	**Median (min-max)/**	***p***	**Median (min-max)/**	***p***	**Median (min-max)/**	***p***
		**Mean ± SD**		**Mean ± SD**		**Mean ± SD**		**Mean ± SD**		**Mean ± SD**	
**Age**											
≤50	13 (31)	221.36 (164.86–292.09)	0.484	508.11 (289.62–5,403.52)	0.591	7.82 (0.30–69.91)	0.419	18.16 (11.08–64.44)	0.468	39.94 (30.98–138.80)	**0.004**
>50	29 (69)	241.80 (133.39–380.97)		500.59 (213.72–6,040.93)		4.26 (1.33–65.08)		24.01 (6.84–147.70)		73.53 (37.61–455.40)	
**Histology**											
Type I	34 (81)	245.59 (159.12–380.97)	0.945	504.48 (213.72–6,040.93)	0.836	4.65 (0.30–65.08)	1.000	22.51 (6.84–147.70)	0.297	58.54 (34.79–455.40)	0.801
Type II	4 (10)	243.52 (133.39–341.24)		484.46 (415.45–56.34)		6.01 (2.91–8.10)		17.34 (11.47–29.42)		63.82 (50.73–136.50)	
Uncertainty	4 (10)										
**Stage**											
G1+G2	31 (74)	248.00 (133.39–380.97)	1.000	500.85 (213.72–6,040.93)	0.408	4.40 (0.30–65.08)	0.432	24.01 (10.37–144.30)	**0.028**	57.75 (34.79–200.30)	0.857
G3	6 (14)	224.80 (214.24–283.37)		532.33 (457.23–992.81)		5.95 (3.78–36.22)		15.09 (6.84–29.42)		58.36 (39.94–113.70)	
Uncertainty	5 (12)										
**Myometrial invasion**											
≤50%	33 (79)	240.65 (159.12–380.97)	0.910	508.11 (213.72–6,040.93)	0.834	4.90 (0.30–69.91)	0.936	20.15 (6.84–68.13)	0.091	54.22 (30.98–291.30)	**0.006**
>50%	8 (19)	245.01 (133.39–341.24)		480.92 (419.63–640.05)		5.28 (2.86–17.13)		33.95 (11.47–144.30)		119.85 (50.73–200.30)	
Uncertainty	1 (2)										
**Metastasis**											
With metastasis	3 (7)	177.26 (133.39–267.52)	0.213	419.63 (289.62–500.59)	0.133	3.99 (0.30–4.26)	0.195	31.92 (29.42–43.98)	0.195	138.80 (136.50–291.30)	**0.004**
Without metastasis	38 (90)	241.22 (159.12–380.97)		509.83 (213.72–6,040.93)		5.61 (1.33–69.91)		20.22 (6.84–144.30)		56.11 (30.98–200.30)	
Uncertainty	1 (2)										

*The statistical analysis of age and myometrial invasion are tested by LSD. The other factors are tested by Mann-Whitney U. p < 0.05 is considered as statistically significant*.

**Figure 4:**
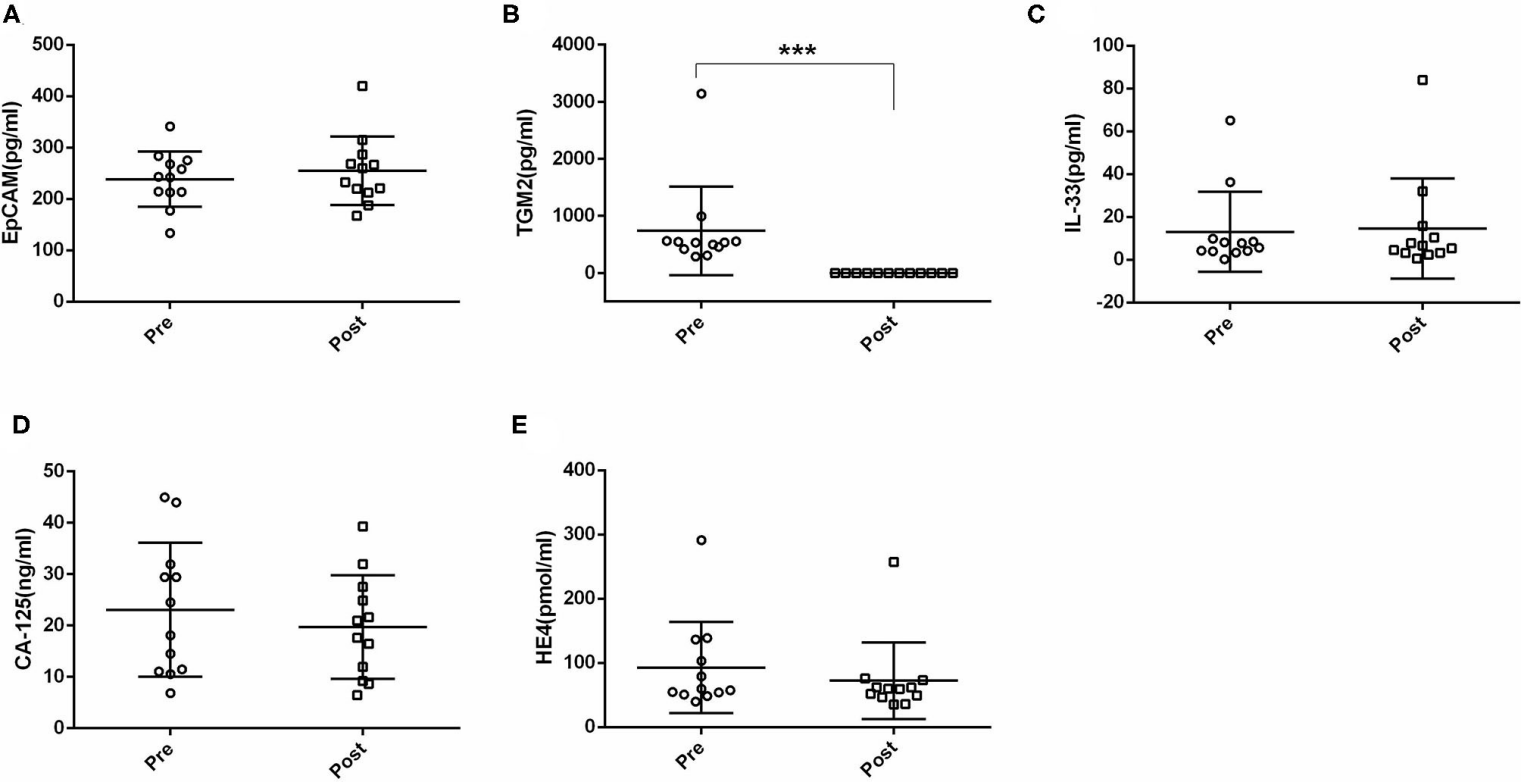
Comparisons of serum EpCAM, TGM2, IL-33, CA-125, and HE4 levels in pre- and post-operative endometrial cancer (EC) patients. **(A)** EpCAM. **(B)** TGM2. **(C)** IL-33. **(D)** CA-125. **(E)** HE4. ***p < 0.001.

Next, we compared the serum EpCAM, TGM2, HE4, CA-125, and IL-33 levels in pre- and post-operative EC patients. Among these markers, the levels of TGM2 were significantly higher in pre-operative than that of post-operative EC patients (*p* < 0.001). However, the difference of EpCAM, HE4, CA-125, and IL-33 was not statistically significant between pre- and post-operative EC patients (*p* = 0.381, *p* = 0.158, *p* = 0.100, *p* = 0.937, respectively) (Figure 4, Table 3).

**Table 3:** Comparison of the levels of EpCAM, TGM2, IL-33, CA125, and HE4 in pre- and post-operated endometrial cancer patients.

	**Pre-operation**	**Post-operation**	***p***
	**Median (min-max)/Mean ± SD**	**Median (min-max)/Mean ± SD**	
EpCAM (pg/ml)	238.51 ± 53.82	254.81 ± 66.77	0.381
TGM2 (pg/ml)	534.85 (289.62–3,143.07)	0 (0–0)	**<0.001**
IL-33 (pg/ml)	6.69 (0.30–65.08)	6.04 (0.59–84.00)	0.937
CA125 (ng/ml)	23.07 ± 13.05	19.07 ± 10.06	0.100
HE4 (pmol/ml)	58.59 (39.94–291.30)	59.79 (35.00–257.60)	0.158

*The statistical analysis of EpCAM and CA125 are tested by paired-samples t-test. TGM2, IL-33, TG, and HE4 are tested by Wilcoxon signed-rank. p < 0.05 is considered as statistically significant*.

## Discussion

In this study, we evaluated the diagnostic and prognostic values of serum EpCAM, TGM2, HE4, CA-125, and IL-33 in EC. All these serum markers levels were found higher in EC patients. The combination of EpCAM and HE4 exhibited the highest AUC value with good sensitivity and specificity for the diagnosis of EC. HE4 and TGM2 seemed to be potential markers for prognostic evaluation of EC.

Nowadays, the fast increasing incidence of EC could be due to the increasing trends in exogenous estrogen use, declined fertility, overweight, and obesity (26). Indeed, we found the significant elevated TG and declined HDL serum levels in the EC group, demonstrating the correlation between obesity and EC. To improve cancer diagnosis, numerous blood biomarkers have been identified for early detection, screening, and monitoring in a range of tumor types (27). However, it still does not reach consensus in the case of EC (6). The first and well-studied EC biomarkers are CA-125 and HE4, which are also included in our study. We discovered that the serum CA-125 and HE4 levels in EC patients were enhanced compared with healthy or benign patients. Only the serum CA-125 levels were associated with the stage of EC, but others reported a probable role of HE4 in predicting the EC stage as well (28–30). The serum HE4 levels were correlated with myometrial invasion and metastasis, which is consisted with the previous studies (28, 30, 31), But, Antonsen et al. also demonstrated that the CA-125 might be useful in the evaluation of histological grade, lymph node metastases, and myometrial invasion (28). Besides, the HE4 alone or combined with CA-125 exhibited a higher EC diagnostic value than the CA-125. These data were in line with the previous report that the diagnostic value of HE4 is better than the CA-125 (32). Of note, it is reported that age greatly affects CA-125 and HE4 levels (33). And in our study, we observed significantly increased serum levels of HE4 but not CA-125 in aged patients. Moreover, HE4 levels were also correlated with menopausal status, obesity, smoking conditions, and creatine values (8). Thus, it would be complicated when dealing with CA-125 and HE4 data clinically. The combination of serum biomarkers with clinical and ultrasound characteristics would greatly improve the efficiency of EC risk management (34).

Of late, emerging novel biomarkers for EC diagnoses have been proposed such as EpCAM, TGM2, IL-33, etc. (19, 20, 35). At first, increased EpCAM expressions were found in almost all carcinomas and known to associated with tumor grading, staging, and prognosis (36). Similarly, overexpressed EpCAM was also observed in advanced EC. The activation of EGFR signaling can initiate EpCAM cleavage, resulting in changes in nanomechanical properties of EC facilitating epithelial-to-mesenchymal transition and cell invasion (37). Conversely, KC Wen et al. reported that EpCAM expressions in tissues were inversely correlated with EC malignancy, suggesting that EpCAM played a paradoxical role in EC (17). Nevertheless, A Torres et al. demonstrated elevated EpCAM levels in blood samples of EC compared with non-EC. Significant higher levels were implicated at stage I/II of EC patients (19). Coincidently, we also identified elevated serum EpCAM levels in EC patients compared with healthy or benign patients. Serum EpCAM levels were higher at stage I+II of EC patients compared with stage III albeit with no significance. Importantly, the EpCAM+HE4 model exhibited the highest AUC value for EC diagnosis. Apart from EpCAM, Torres et al. also first identified another marker TGM2 for prognostic evaluation of EC (19). In our study, we also estimated increased TGM2 levels in EC patients, and its levels were not correlated with clinicopathological factors. It is noteworthy that, we also estimated the prognostic values of biomarkers by measuring their levels in pre- and post-operative EC patients. Beyond our expectations, the serum TGM2 levels declined to zero post-operatively with multiple assays, indicating it might be a good marker for follow-up of EC. Notably, the average serum TGM2 levels were higher in the EC group compared with the benign and healthy group. The occasionally higher TGM2 levels in healthy controls make no significant difference between the healthy and EC group. However, Torres et al. reported significantly elevated TGM2 levels when compared with the healthy and benign group (19). They detected plasma TGM2 levels, but we tested its serum levels. This might be one of the underlying reasons. Other factors that might influence TGM2 levels needs further evaluation.

In recent years, growing studies have demonstrated the potential role of IL-33 in the diagnosis of breast, lung, and gastric cancer (38–40). Few studies of IL-33 were reported on gynecologic cancer. Zeng et al. first reported that the serum IL-33 levels were remarkably increased in EC patients than that of healthy controls, and correlated with EC prognosis (20). However, patients with myoma or endometritis were not included and the follow-up period was <18 months in their study (20). Besides, the immunohistochemical analysis identified a positive correlation of IL-33 levels and grades in both serous and mucinous epithelial ovarian tumors (21). In our study, we also found the serum IL-33 levels were significantly increased in EC patients when compared with the healthy group. But IL-33 levels were not correlated with stages, histological grades, and myometrial invasion of EC possibly due to the small sample size of EC patients.

In conclusion, our study showed that serum EpCAM, TGM2, HE-4, CA-125, and IL-33 levels were significantly higher in EC patients. Among them, the EpCAM+HE-4 model seemed to be more effective than CA-125 or HE4, providing a more accurate basis for clinical diagnosis. We also confirmed that the TGM2 and HE4 may be an important prognostic factor for EC. Larger population investigations are necessary to validate our observations.

## Data Availability Statement

All datasets presented in this study are included in the article/Supplementary Material.

## Ethics Statement

The studies involving human participants were reviewed and approved by The Clinical Research Ethics Committee, The Affiliated Hospital Of Xuzhou Medical University. The patients/participants provided their written informed consent to participate in this study. Written informed consent was obtained from the individual(s) for the publication of any potentially identifiable images or data included in this article.

## Author Contributions

TL, LL, and PM designed the study. TL wrote the manuscript. CM and ZW analyzed the data and interpreted results. CM, YW, YL, HX, and SL worked on patient serum and clinical data collection. YM, LL, and BG reviewed and revised the manuscript. All authors contributed to the interpretation of the findings, critically commented on the manuscript, and approved the submitted version.

## Conflict of Interest

The authors declare that the research was conducted in the absence of any commercial or financial relationships that could be construed as a potential conflict of interest.
