# Supplementary material for: Diagnostic and Prognostic Values of Serum EpCAM, TGM2, and HE4 Levels in Endometrial Cancer
Source: Front Oncol. 2020 Sep 4;10:1697. doi: 10.3389/fonc.2020.01697 (PMC7498689; doi:10.3389/fonc.2020.01697)
Supplement: Supplementary file 1 [file Data_Sheet_1.docx]

**Supplied Table 1.** Comparison of clinical features between benign and endometrial cancer (EC) group.

|  | Benign n (%) | EC n (%) | p |
| --- | --- | --- | --- |
| Menopusal status |  |  |  |
| Premenopausal | 36(88) | 24(57) | **<0.001** |
| Postmenopausal | 5(12) | 18(43) |  |
| Diabetes |  |  |  |
| Yes | 2(5) | 3(7) | 1.000 |
| No | 39(95) | 39(93) |  |
| Hypertension |  |  |  |
| Yes | 4(10) | 24(57) | **<0.001** |
| No | 37(90) | 18(43) |  |
| Menarche (years old) |  |  |  |
| 11 | 1(2) | 0(0) | **0.005** |
| 12 | 7(17) | 3(7) |  |
| 13 | 12(29) | 7(17) |  |
| 14 | 9(22) | 9(21) |  |
| 15 | 4(10) | 8(19) |  |
| 16 | 5(12) | 5(12) |  |
| 17 | 0(0) | 3(7) |  |
| 18 | 0(0) | 1(2) |  |
| 19 | 0(0) | 2(5) |  |
| uncertainty | 3(7) | 4(10) |  |
| Term |  |  |  |
| 0 | 1(2) | 1(2) | **0.001** |
| 1 | 22(54) | 6(14) |  |
| 2 | 11(27) | 23(55) |  |
| 3 | 2(5) | 7(17) |  |
| 4 | 3(7) | 2(5) |  |
| 5 | 0(0) | 1(2) |  |
| uncertainty | 2(5) | 2(5) |  |
| Prematurity |  |  |  |
| 0 | 39(95) | 40(95) | 0.317 |
| 1 | 1(2) | 0(0) |  |
| uncertainty | 1(2) | 2(5) |  |
| Abortion |  |  |  |
| 0 | 12(29) | 25(60) | **0.009** |
| 1 | 18(44) | 9(21) |  |
| 2 | 6(15) | 4(10) |  |
| 3 | 4(10) | 2(5) |  |
| uncertainty | 1(2) | 2(5) |  |
| Parity |  |  |  |
| 0 | 1(2) | 2(5) | **0.004** |
| 1 | 22(54) | 6(14) |  |
| 2 | 11(27) | 23(55) |  |
| 3 | 4(10) | 7(17) |  |
| 4 | 1(2) | 1(2) |  |
| 5 | 0(0) | 1(2) |  |
| uncertainty | 2(5) | 2(5) |  |
| Ligation |  |  |  |
| Yes | 6(15) | 10(24) | 0.289 |
| No | 35(85) | 32(76) |  |

The statistical analysis of menopausal status, diabetes, hypertension, and ligation are tested by chi-square test. The statistical analysis of menarche, term, prematurity, abortion, and survival are tested by Mann-Whitney U.

**Supplied Table 2.** ROC curves results, likelihood ratio and predictive values of EpCAM, TGM2, IL-33, CA-125 and HE4 in the diagnosis of EC compared with non-EC (including healthy and benign group).

|  | Cutoff value | Sensitivity (%) | Speciﬁcity (%) | Youden Index | ROC AUC | 95% CI | PLR (%) | NLR (%) | PPV (%) | NPV (%) |
| --- | --- | --- | --- | --- | --- | --- | --- | --- | --- | --- |
| EpCAM | 204.90 | 83.33 | 64.29 | 0.476 | 0.745 | 0.656-0.834 | 58.33 | 6.48 | 53.85 | 88.52 |
| TGM2 | 359.71 | 90.48 | 41.67 | 0.321 | 0.646 | 0.552-0.740 | 38.78 | 5.71 | 43.68 | 89.74 |
| IL-33 | 1.29 | 97.62 | 22.62 | 0.202 | 0.569 | 0.467-0.671 | 31.54 | 2.63 | 38.68 | 95.00 |
| CA-125 | 17.07 | 73.81 | 67.86 | 0.417 | 0.722 | 0.629-0.815 | 57.41 | 9.65 | 53.45 | 83.82 |
| HE4 | 49.32 | 71.43 | 84.52 | 0.560 | 0.827 | 0.749-0.905 | 115.4 | 8.45 | 69.77 | 85.54 |
| EpCAM+HE4 |  | 92.86 | 69.05 | 0.619 | 0.874 | 0.809-0.939 | 75.00 | 2.59 | 60.00 | 95.08 |
| CA-125+HE4 |  | 73.81 | 79.76 | 0.536 | 0.830 | 0.753-0.906 | 91.18 | 8.21 | 64.58 | 85.90 |
| EpCAM+CA-125 |  | 95.24 | 59.52 | 0.548 | 0.805 | 0.729-0.881 | 58.82 | 2.00 | 54.05 | 96.15 |
| EpCAM+CA-125+HE4 |  | 83.33 | 77.38 | 0.607 | 0.881 | 0.819-0.943 | 92.11 | 5.38 | 64.81 | 90.28 |

**Supplied Table 3.** ROC curves results, likelihood ratio and predictive values of EpCAM, TGM2, IL-33, CA-125 and HE4 in the diagnosis of EC compared with healthy group.

|  | | Cutoff value | | Sensitivity (%) | | Speciﬁcity (%) | | Youden Index | | ROC AUC | | 95% CI | | PLR (%) | NLR (%) | | PPV (%) | | NPV (%) | |  |
| --- | --- | --- | --- | --- | --- | --- | --- | --- | --- | --- | --- | --- | --- | --- | --- | --- | --- | --- | --- | --- | --- |
| EpCAM | 204.90 | | 83.33 | | 69.77 | | 0.531 | | 0.804 | | 0.701-0.898 | | 244.19 | | | 23.58 | | 71.43 | | 80.56 | |
| TGM2 | 263.73 | | 97.62 | | 27.91 | | 0.255 | | 0.550 | | 0.423-0.677 | | 129.18 | | | 8.14 | | 56.94 | | 92.31 | |
| IL-33 | 2.37 | | 85.71 | | 53.49 | | 0.392 | | 0.679 | | 0.562-0.797 | | 175.81 | | | 25.48 | | 64.29 | | 79.31 | |
| CA-125 | 17.07 | | 73.81 | | 72.09 | | 0.459 | | 0.760 | | 0.657-0.862 | | 252.33 | | | 34.66 | | 72.09 | | 73.81 | |
| HE4 | 45.18 | | 80.95 | | 90.70 | | 0.717 | | 0.929 | | 0.877-0.981 | | 830.23 | | | 20.04 | | 89.47 | | 82.98 | |
| EpCAM+HE4 |  | | 92.86 | | 93.02 | | 0.859 | | 0.957 | | 0.911-1.000 | | 1269.77 | | | 7.33 | | 92.86 | | 93.02 | |
| CA-125+HE4 |  | | 80.95 | | 93.02 | | 0.740 | | 0.934 | | 0.884-0.983 | | 327.60 | | | 5.78 | | 80.95 | | 93.02 | |
| EpCAM+CA-125 |  | | 95.24 | | 72.09 | | 0.673 | | 0.875 | | 0.803-0.948 | | 325.58 | | | 6.30 | | 76.92 | | 93.94 | |
| EpCAM+CA-125+HE4 | |  | 92.86 | | 93.02 | | 0.859 | | 0.961 | | 0.918-1.000 | | 1269.77 | | | 7.33 | | 92.86 | | 93.02 | |

**Supplied Table 4.** ROC curves results, likelihood ratio and predictive values of EpCAM, TGM2, IL-33, CA-125 and HE4 in the diagnosis of EC compared with benign group.

|  | Cutoff value | Sensitivity (%) | Speciﬁcity (%) | Youden Index | ROC AUC | 95% CI | PLR (%) | NLR (%) | PPV (%) | NPV (%) |
| --- | --- | --- | --- | --- | --- | --- | --- | --- | --- | --- |
| EpCAM | 204.18 | 83.33 | 58.54 | 0.419 | 0.683 | 0.566-0.800 | 210.9 | 29.88 | 67.31 | 77.42 |
| TGM2 | 455.75 | 69.05 | 75.61 | 0.447 | 0.747 | 0.639-0.856 | 297.07 | 42.96 | 74.36 | 70.45 |
| IL-33 | 15.28 | 16.67 | 95.12 | 0.118 | 0.454 | 0.326-0.581 | 358.54 | 91.93 | 77.78 | 52.7 |
| CA-125 | 16.72 | 73.81 | 63.41 | 0.372 | 0.682 | 0.567-0.798 | 211.71 | 43.34 | 67.39 | 70.27 |
| HE4 | 54.1 | 61.9 | 78.05 | 0.4 | 0.72 | 0.606-0.833 | 295.93 | 51.22 | 74.29 | 66.67 |
| EpCAM+HE4 |  | 64.29 | 80.49 | 0.448 | 0.787 | 0.690-0.884 | 345.73 | 46.56 | 77.14 | 68.75 |
| CA-125+HE4 |  | 40.48 | 100 | 0.405 | 0.721 | 0.609-0.832 | 17.52 | 0 | 40.48 | 100 |
| EpCAM+CA-125 |  | 88.1 | 56.1 | 0.442 | 0.731 | 0.622-0.840 | 210.57 | 22.27 | 67.27 | 82.14 |
| EpCAM+CA-125+HE4 |  | 71.43 | 78.05 | 0.495 | 0.797 | 0.703-0.892 | 341.46 | 38.41 | 76.92 | 72.73 |

**Supplied Table 5.** EpCAM, TGM2, HE4, CA-125, and IL-33 measured in serum samples from 42 patients with endometrial cancer in relation to clinicopathological factors of Grade 1 compared with non-EC (including healthy and benign group).

|  | Cutoff value | Sensitivity (%) | Speciﬁcity (%) | Youden Index | ROC AUC | 95% CI | PLR (%) | NLR (%) | PPV (%) | NPV (%) |
| --- | --- | --- | --- | --- | --- | --- | --- | --- | --- | --- |
| EpCAM | 214.09 | 100.00 | 67.86 | 0.679 | 0.792 | 0.684-0.899 | 1.59 | 0 | 18.18 | 100.00 |
| TGM2 | 455.97 | 100.00 | 58.33 | 0.583 | 0.710 | 0.589-0.832 | 1.22 | 0 | 14.63 | 100.00 |
| IL-33 | 3.76 | 100.00 | 42.86 | 0.429 | 0.621 | 0.455-0.787 | 0.89 | 0 | 11.11 | 100.00 |
| CA-125 | 17.07 | 50.00 | 67.86 | 0.179 | 0.508 | 0.278-0.737 | 0.79 | 0.38 | 10.00 | 95.00 |
| HE4 | 48.27 | 83.33 | 78.57 | 0.619 | 0.851 | 0.712-0.991 | 1.98 | 0.11 | 21.74 | 98.51 |
| EpCAM+HE4 |  | 100.00 | 82.14 | 0.821 | 0.911 | 0.840-0.981 | 2.86 | 0 | 28.57 | 100.00 |
| CA-125+HE4 |  | 66.67 | 89.29 | 0.560 | 0.833 | 0.683-0.984 | 33.77 | 2.03 | 66.67 | 89.29 |
| EpCAM+CA-125 |  | 100.00 | 61.90 | 0.619 | 0.750 | 0.639-0.861 | 1.34 | 0 | 15.79 | 100.00 |
| EpCAM+CA-125+HE4 |  | 100.00 | 80.95 | 0.810 | 0.907 | 0.835-0.978 | 2.68 | 0 | 27.27 | 100.00 |

**Supplied Table 6.** EpCAM, TGM2, HE4, CA-125, and IL-33 measured in serum samples from 42 patients with endometrial cancer in relation to clinicopathological factors of Grade 2 compared with non-EC (including healthy and benign group).

|  | Cutoff value | Sensitivity (%) | Speciﬁcity (%) | Youden Index | ROC AUC | 95% CI | PLR (%) | NLR (%) | PPV (%) | NPV (%) |
| --- | --- | --- | --- | --- | --- | --- | --- | --- | --- | --- |
| EpCAM | 247.83 | 77.78 | 86.90 | 0.647 | 0.820 | 0.696-0.944 | 6.82 | 0.29 | 38.89 | 97.33 |
| TGM2 | 277.50 | 100.00 | 33.33 | 0.333 | 0.598 | 0.443-0.753 | 1.72 | 0 | 13.85 | 100.00 |
| IL-33 | 0.15 | 100.00 | 16.67 | 0.167 | 0.517 | 0.321-0.713 | 1.07 | 1.53 | 9.09 | 87.50 |
| CA-125 | 17.33 | 88.89 | 67.86 | 0.567 | 0.788 | 0.665-0.912 | 3.17 | 0.19 | 22.86 | 98.28 |
| HE4 | 50.19 | 66.67 | 85.71 | 0.524 | 0.816 | 0.674-0.959 | 5.36 | 0.45 | 33.33 | 96.00 |
| EpCAM+HE4 |  | 100.00 | 72.62 | 0.726 | 0.881 | 0.796-0.966 | 4.19 | 0 | 28.13 | 100.00 |
| CA-125+HE4 |  | 77.78 | 75.00 | 0.528 | 0.813 | 0.669-0.958 | 150.77 | 14.36 | 77.78 | 75.00 |
| EpCAM+CA-125 |  | 100.00 | 73.81 | 0.738 | 0.899 | 0.824-0.975 | 4.38 | 0.00 | 29.03 | 100.00 |
| EpCAM+CA-125+HE4 |  | 100.00 | 66.67 | 0.667 | 0.893 | 0.802-0.984 | 3.44 | 0 | 24.32 | 100.00 |

**Supplied Table 7.** EpCAM, TGM2, HE4, CA-125, and IL-33 measured in serum samples from 42 patients with endometrial cancer in relation to clinicopathological factors of Grade 3 compared with non-EC (including healthy and benign group).

|  | Cutoff value | Sensitivity (%) | Speciﬁcity (%) | Youden Index | ROC AUC | 95% CI | PLR (%) | NLR (%) | PPV (%) | NPV (%) |
| --- | --- | --- | --- | --- | --- | --- | --- | --- | --- | --- |
| EpCAM | 206.97 | 81.82 | 64.29 | 0.461 | 0.739 | 0.624-0.854 | 15.71 | 1.94 | 37.50 | 93.10 |
| TGM2 | 359.71 | 90.91 | 41.67 | 0.326 | 0.641 | 0.534-0.749 | 10.69 | 1.50 | 28.99 | 94.59 |
| IL-33 | 1.29 | 100.00 | 22.62 | 0.226 | 0.561 | 0.438-0.684 | 8.86 | 0 | 25.29 | 100.00 |
| CA-125 | 17.79 | 72.73 | 69.05 | 0.418 | 0.732 | 0.618-0.845 | 16.12 | 2.71 | 38.10 | 90.63 |
| HE4 | 49.32 | 77.27 | 84.52 | 0.618 | 0.854 | 0.761-0.946 | 34.25 | 1.84 | 56.67 | 93.42 |
| EpCAM+HE4 |  | 86.36 | 78.57 | 0.649 | 0.895 | 0.823-0.967 | 27.65 | 1.19 | 51.35 | 95.65 |
| CA-125+HE4 |  | 81.82 | 79.76 | 0.616 | 0.861 | 0.774-0.948 | 221.83 | 12.51 | 81.82 | 79.76 |
| EpCAM+CA-125 |  | 95.45 | 59.52 | 0.550 | 0.798 | 0.705-0.891 | 16.18 | 0.52 | 38.18 | 98.04 |
| EpCAM+CA-125+HE4 |  | 90.91 | 73.81 | 0.647 | 0.902 | 0.836-0.968 | 23.81 | 0.84 | 47.62 | 96.88 |

**Supplied Table 8.** EpCAM, TGM2, HE4, CA-125, and IL-33 measured in serum samples from 42 patients with endometrial cancer in relation to clinicopathological factors of Grade 1 and Grade 2 compared with non-EC (including healthy and benign group).

|  | Cutoff value | Sensitivity (%) | Speciﬁcity (%) | Youden Index | ROC AUC | 95% CI | PLR (%) | NLR (%) | PPV (%) | NPV (%) |
| --- | --- | --- | --- | --- | --- | --- | --- | --- | --- | --- |
| EpCAM | 208.57 | 93.33 | 64.29 | 0.576 | 0.809 | 0.713-0.904 | 8.33 | 0.33 | 31.82 | 98.18 |
| TGM2 | 277.50 | 100.00 | 33.33 | 0.333 | 0.643 | 0.523-0.763 | 4.78 | 0 | 21.13 | 100.00 |
| IL-33 | 0.15 | 100.00 | 16.67 | 0.167 | 0.559 | 0.413-0.704 | 3.83 | 0 | 17.65 | 100.00 |
| CA-125 | 17.07 | 73.33 | 67.86 | 0.412 | 0.676 | 0.533-0.819 | 7.28 | 1.25 | 28.95 | 93.44 |
| HE4 | 46.76 | 80.00 | 72.62 | 0.526 | 0.830 | 0.724-0.937 | 9.32 | 0.88 | 34.29 | 95.31 |
| EpCAM+HE4 |  | 100.00 | 72.62 | 0.726 | 0.893 | 0.827-0.959 | 11.65 | 0 | 39.47 | 100.00 |
| CA-125+HE4 |  | 66.67 | 83.33 | 0.500 | 0.821 | 0.712-0.931 | 64.00 | 6.40 | 66.67 | 83.33 |
| EpCAM+CA-125 |  | 100.00 | 61.90 | 0.619 | 0.840 | 0.757-0.922 | 8.37 | 0 | 31.91 | 100.00 |
| EpCAM+CA-125+HE4 |  | 86.67 | 80.95 | 0.676 | 0.898 | 0.83-0.966 | 14.51 | 0.53 | 44.83 | 97.14 |

**Supplied Table 9.** EpCAM, TGM2, HE4, CA-125, and IL-33 measured in serum samples from 42 patients with endometrial cancer in relation to clinicopathological factors of Grade 1 compared with healthy group.

|  | Cutoff value | Sensitivity (%) | Speciﬁcity (%) | Youden Index | ROC AUC | 95% CI | PLR (%) | NLR (%) | PPV (%) | NPV (%) |
| --- | --- | --- | --- | --- | --- | --- | --- | --- | --- | --- |
| EpCAM | 214.09 | 100.00 | 76.74 | 0.767 | 0.868 | 0.763-0.974 | 8.37 | 0 | 37.50 | 100.00 |
| TGM2 | 437.47 | 100.00 | 41.86 | 0.419 | 0.601 | 0.427-0.774 | 3.35 | 0 | 19.35 | 100.00 |
| IL-33 | 3.76 | 100.00 | 62.79 | 0.628 | 0.729 | 0.588-0.869 | 5.23 | 0 | 27.27 | 100.00 |
| CA-125 | 11.07 | 83.33 | 41.86 | 0.252 | 0.566 | 0.332-0.800 | 2.79 | 0.78 | 16.67 | 94.74 |
| HE4 | 39.93 | 100.00 | 81.40 | 0.814 | 0.961 | 0.898-1.000 | 10.47 | 0 | 42.86 | 100.00 |
| EpCAM+HE4 |  | 100.00 | 97.67 | 0.977 | 0.988 | 0.962-1.000 | 83.72 | 0 | 85.71 | 100.00 |
| CA-125+HE4 |  | 100.00 | 81.40 | 0.814 | 0.957 | 0.892-1..000 | +∞ | 9.14 | 100.00 | 81.40 |
| EpCAM+CA-125 |  | 100.00 | 72.09 | 0.721 | 0.845 | 0.730-0.960 | 6.98 | 0 | 33.33 | 100.00 |
| EpCAM+CA-125+HE4 |  | 100.00 | 97.67 | 0.977 | 0.988 | 0.962-1.000 | 83.72 | 0 | 85.71 | 100.00 |

**Supplied Table 10.** EpCAM, TGM2, HE4, CA-125, and IL-33 measured in serum samples from 42 patients with endometrial cancer in relation to clinicopathological factors of Grade 2 compared with healthy group.

|  | Cutoff value | Sensitivity (%) | Speciﬁcity (%) | Youden Index | ROC AUC | 95% CI | PLR(%) | NLR(%) | PPV(%) | NPV(%) |
| --- | --- | --- | --- | --- | --- | --- | --- | --- | --- | --- |
| EpCAM | 247.32 | 77.78 | 95.35 | 0.731 | 0.876 | 0.751-1.000 | 73.26 | 1.02 | 77.78 | 95.35 |
| TGM2 | 263.73 | 100.00 | 27.91 | 0.279 | 0.509 | 0.330-0.688 | 6.08 | 0 | 22.50 | 100.00 |
| IL-33 | 0.15 | 100.00 | 32.56 | 0.326 | 0.628 | 0.457-0.799 | 6.50 | 0 | 23.68 | 100.00 |
| CA-125 | 17.33 | 88.89 | 72.09 | 0.610 | 0.817 | 0.694-0.939 | 13.95 | 0.68 | 40.00 | 96.88 |
| HE4 | 37.58 | 100.00 | 72.09 | 0.721 | 0.933 | 0.853-1.000 | 15.70 | 0 | 42.86 | 100.00 |
| EpCAM+HE4 |  | 100.00 | 97.67 | 0.977 | 0.987 | 0.960-1.000 | 188.37 | 0 | 90.00 | 100.00 |
| CA-125+HE4 |  | 77.78 | 97.67 | 0.755 | 0.933 | 0.852-1.000 | 63.64 | 0.43 | 77.78 | 97.67 |
| EpCAM+CA-125 |  | 100.00 | 83.72 | 0.837 | 0.959 | 0.907-1.000 | 26.91 | 0 | 56.25 | 100.00 |
| EpCAM+CA-125+HE4 |  | 100.00 | 93.02 | 0.930 | 0.982 | 0.952-1.000 | 62.79 | 0 | 75.00 | 100.00 |

**Supplied Table 11.** EpCAM, TGM2, HE4, CA-125, and IL-33 measured in serum samples from 42 patients with endometrial cancer in relation to clinicopathological factors of Grade 3 compared with healthy group.

|  | Cutoff value | Sensitivity (%) | Speciﬁcity (%) | Youden Index | ROC AUC | 95% CI | PLR (%) | NLR (%) | PPV (%) | NPV (%) |
| --- | --- | --- | --- | --- | --- | --- | --- | --- | --- | --- |
| EpCAM | 206.97 | 81.82 | 69.77 | 0.516 | 0.796 | 0.676-0.916 | 70.84 | 6.82 | 58.06 | 88.24 |
| TGM2 | 359.71 | 90.91 | 34.88 | 0.258 | 0.548 | 0.409-0.686 | 36.54 | 6.82 | 41.67 | 88.24 |
| IL-33 | 2.37 | 86.36 | 53.49 | 0.399 | 0.670 | 0.542-0.789 | 48.60 | 6.67 | 48.72 | 88.46 |
| CA-125 | 17.79 | 72.73 | 74.42 | 0.471 | 0.768 | 0.652-0.885 | 74.42 | 9.59 | 59.26 | 84.21 |
| HE4 | 49.11 | 77.27 | 100.00 | 0.773 | 0.947 | 0.894-1.000 | +∞ | 5.95 | 100.00 | 89.58 |
| EpCAM+HE4 |  | 95.45 | 93.02 | 0.885 | 0.971 | 0.932-1.000 | 358.14 | 1.28 | 87.50 | 97.56 |
| CA-125+HE4 |  | 86.36 | 95.35 | 0.817 | 0.955 | 0.905-1.000 | 302.27 | 2.33 | 86.36 | 95.35 |
| EpCAM+CA-125 |  | 95.45 | 72.09 | 0.675 | 0.866 | 0.778-0.954 | 89.53 | 1.65 | 63.64 | 96.88 |
| EpCAM+CA-125+HE4 |  | 95.45 | 93.02 | 0.885 | 0.979 | 0.950-1.000 | 358.14 | 1.28 | 87.50 | 97.56 |

**Supplied Table 12.** EpCAM, TGM2, HE4, CA-125, and IL-33 measured in serum samples from 42 patients with endometrial cancer in relation to clinicopathological factors of Grade I and Grade 2 compared with healthy group.

|  | Cutoff value | Sensitivity (%) | Speciﬁcity (%) | Youden Index | ROC AUC | 95% CI | PLR (%) | NLR (%) | PPV (%) | NPV (%) |
| --- | --- | --- | --- | --- | --- | --- | --- | --- | --- | --- |
| EpCAM | 214.09 | 86.67 | 76.74 | 0.634 | 0.873 | 0.777-0.968 | 45.35 | 2.11 | 56.52 | 94.29 |
| TGM2 | 263.73 | 100.00 | 27.91 | 0.279 | 0.546 | 0.395-0.697 | 16.88 | 0 | 32.61 | 100.00 |
| IL-33 | 2.84 | 80.00 | 55.81 | 0.358 | 0.668 | 0.529-0.807 | 60.61 | 5.68 | 57.14 | 88.89 |
| CA-125 | 17.07 | 73.33 | 72.09 | 0.454 | 0.716 | 0.569-0.863 | 31.98 | 4.50 | 47.83 | 88.57 |
| HE4 | 37.58 | 100.00 | 72.09 | 0.721 | 0.944 | 0.886-1.000 | 43.60 | 0 | 55.56 | 100.00 |
| EpCAM+HE4 |  | 100.00 | 97.67 | 0.977 | 0.988 | 0.962-1.000 | 523.26 | 0 | 93.75 | 100.00 |
| CA-125+HE4 |  | 80.00 | 93.02 | 0.730 | 0.943 | 0.883-1.000 | 139.53 | 2.62 | 80.00 | 93.02 |
| EpCAM+CA-125 |  | 100.00 | 72.09 | 0.721 | 0.913 | 0.842-0.984 | 43.60 | 0 | 55.56 | 100.00 |
| EpCAM+CA-125+HE4 |  | 100.00 | 93.02 | 0.930 | 0.984 | 0.959-1.000 | 174.42 | 0 | 83.33 | 100.00 |

**Supplied Table 13.** EpCAM, TGM2, HE4, CA-125, and IL-33 measured in serum samples from 42 patients with endometrial cancer in relation to clinicopathological factors of Grade 1 compared with benign group.

|  | Cutoff value | Sensitivity (%) | Speciﬁcity (%) | Youden Index | ROC AUC | 95% CI | PLR (%) | NLR (%) | PPV (%) | NPV (%) |
| --- | --- | --- | --- | --- | --- | --- | --- | --- | --- | --- |
| EpCAM | 208.54 | 100.00 | 58.54 | 0.585 | 0.711 | 0.561-0.861 | 5.16 | 0 | 26.09 | 100.00 |
| TGM2 | 455.97 | 100.00 | 75.61 | 0.756 | 0.825 | 0.709-0.941 | 8.78 | 0 | 37.50 | 100.00 |
| IL-33 | 3.68 | 100.00 | 21.95 | 0.220 | 0.508 | 0.270-0.747 | 2.74 | 0 | 15.79 | 100.00 |
| CA-125 | 16.72 | 50.00 | 63.41 | 0.134 | 0.447 | 0.198-0.697 | 2.93 | 1.69 | 16.67 | 89.66 |
| HE4 | 55.74 | 66.67 | 78.05 | 0.447 | 0.736 | 0.508-0.963 | 6.50 | 0.91 | 30.77 | 94.12 |
| EpCAM+HE4 |  | 100.00 | 65.85 | 0.659 | 0.829 | 0.698-0.960 | 6.27 | 0 | 30.00 | 100.00 |
| CA-125+HE4 |  | 66.67 | 78.05 | 0.447 | 0.703 | 0.456-0.951 | 76.47 | 10.75 | 66.67 | 78.05 |
| EpCAM+CA-125 |  | 100.00 | 51.22 | 0.512 | 0.650 | 0.499-0.802 | 4.39 | 0.00 | 23.08 | 100.00 |
| EpCAM+CA-125+HE4 |  | 100.00 | 63.41 | 0.634 | 0.821 | 0.687-0.955 | 5.85 | 0 | 28.57 | 100.00 |

**Supplied Table 14.** EpCAM, TGM2, HE4, CA-125, and IL-33 measured in serum samples from 42 patients with endometrial cancer in relation to clinicopathological factors of Grade 2 compared with benign group.

|  | Cutoff value | Sensitivity (%) | Speciﬁcity (%) | Youden Index | ROC AUC | 95% CI | PLR (%) | NLR (%) | PPV (%) | NPV (%) |
| --- | --- | --- | --- | --- | --- | --- | --- | --- | --- | --- |
| EpCAM | 247.83 | 77.78 | 78.05 | 0.558 | 0.762 | 0.613-0.910 | 17.07 | 1.37 | 43.75 | 94.12 |
| TGM2 | 277.50 | 100.00 | 39.02 | 0.390 | 0.691 | 0.524-0.858 | 7.90 | 0 | 26.47 | 100.00 |
| IL-33 | 11.87 | 22.22 | 90.24 | 0.125 | 0.401 | 0.152-0.650 | 10.98 | 4.15 | 33.33 | 84.09 |
| CA-125 | 16.97 | 88.89 | 63.41 | 0.523 | 0.759 | 0.602-0.915 | 11.71 | 0.84 | 34.78 | 96.30 |
| HE4 | 50.19 | 66.67 | 70.73 | 0.374 | 0.694 | 0.476-0.911 | 10.98 | 2.27 | 33.33 | 90.63 |
| EpCAM+HE4 |  | 100.00 | 46.34 | 0.463 | 0.770 | 0.612-0.928 | 8.98 | 0 | 29.03 | 100.00 |
| CA-125+HE4 |  | 44.44 | 97.56 | 0.420 | 0.688 | 0.467-0.910 | 8.89 | 0.28 | 44.44 | 97.56 |
| EpCAM+CA-125 |  | 100.00 | 63.41 | 0.634 | 0.837 | 0.719-0.956 | 13.17 | 0 | 37.50 | 100.00 |
| EpCAM+CA-125+HE4 |  | 55.56 | 95.12 | 0.507 | 0.799 | 0.635-0.964 | 54.88 | 2.25 | 71.43 | 90.70 |

**Supplied Table 15.** EpCAM, TGM2, HE4, CA-125, and IL-33 measured in serum samples from 42 patients with endometrial cancer in relation to clinicopathological factors of Grade 3 compared with benign group.

|  | Cutoff value | Sensitivity (%) | Speciﬁcity (%) | Youden Index | ROC AUC | 95% CI | PLR (%) | NLR (%) | PPV (%) | NPV (%) |
| --- | --- | --- | --- | --- | --- | --- | --- | --- | --- | --- |
| EpCAM | 206.25 | 81.82 | 58.54 | 0.404 | 0.680 | 0.544-0.815 | 56.81 | 8.94 | 51.43 | 85.71 |
| TGM2 | 455.75 | 68.18 | 75.61 | 0.438 | 0.739 | 0.618-0.861 | 80.49 | 12.12 | 60.00 | 81.58 |
| IL-33 | 9.61 | 27.27 | 82.93 | 0.102 | 0.446 | 0.287-0.605 | 45.99 | 25.25 | 46.15 | 68.00 |
| CA-125 | 20.10 | 63.64 | 73.17 | 0.368 | 0.693 | 0.554-0.832 | 68.29 | 14.31 | 56.00 | 78.95 |
| HE4 | 54.10 | 68.18 | 78.05 | 0.462 | 0.756 | 0.617-0.895 | 89.43 | 11.74 | 62.50 | 82.05 |
| EpCAM+HE4 |  | 59.09 | 92.68 | 0.518 | 0.815 | 0.701-0.929 | 232.52 | 12.71 | 81.25 | 80.85 |
| CA-125+HE4 |  | 45.45 | 100.00 | 0.455 | 0.763 | 0.629-0.896 | 15.72 | 0 | 45.45 | 100.00 |
| EpCAM+CA-125 |  | 90.91 | 53.66 | 0.446 | 0.726 | 0.601-0.852 | 56.48 | 4.88 | 51.28 | 91.67 |
| EpCAM+CA-125+HE4 |  | 63.64 | 92.68 | 0.563 | 0.822 | 0.710-0.933 | 250.41 | 11.30 | 82.35 | 82.61 |

**Supplied Table 16.** EpCAM, TGM2, HE4, CA-125, and IL-33 measured in serum samples from 42 patients with endometrial cancer in relation to clinicopathological factors of Grade 1 and Grade2 compared with benign group.

|  | Cutoff value | Sensitivity (%) | Speciﬁcity (%) | Youden Index | ROC AUC | 95% CI | PLR (%) | NLR (%) | PPV (%) | NPV (%) |
| --- | --- | --- | --- | --- | --- | --- | --- | --- | --- | --- |
| EpCAM | 207.86 | 93.33 | 58.54 | 0.519 | 0.741 | 0.613-0.870 | 30.13 | 1.52 | 45.16 | 96.00 |
| TGM2 | 455.97 | 73.33 | 75.61 | 0.489 | 0.745 | 0.613-0.876 | 40.24 | 4.72 | 52.38 | 88.57 |
| IL-33 | 11.87 | 20.00 | 90.24 | 0.102 | 0.444 | 0.258-0.630 | 27.44 | 11.87 | 42.86 | 75.51 |
| CA-125 | 16.72 | 73.33 | 63.41 | 0.367 | 0.634 | 0.466-0.802 | 26.83 | 5.63 | 42.31 | 86.67 |
| HE4 | 50.19 | 66.67 | 70.73 | 0.374 | 0.711 | 0.545-0.876 | 30.49 | 6.31 | 45.45 | 85.29 |
| EpCAM+HE4 |  | 100.00 | 46.34 | 0.463 | 0.793 | 0.672-0.914 | 24.94 | 0 | 40.54 | 100.00 |
| CA-125+HE4 |  | 40.00 | 97.56 | 0.376 | 0.694 | 0.523-0.865 | 9.52 | 0.36 | 40.00 | 97.56 |
| EpCAM+CA-125 |  | 100.00 | 51.22 | 0.512 | 0.763 | 0.639-0.886 | 27.44 | 0 | 42.86 | 100.00 |
| EpCAM+CA-125+HE4 |  | 73.33 | 78.05 | 0.514 | 0.808 | 0.686-0.930 | 44.72 | 4.57 | 55.00 | 88.89 |
